# International Practical Temperature Scale of 1948: Text Revision of 1960

**DOI:** 10.6028/jres.065A.017

**Published:** 1961-06-01

**Authors:** H. F. Stimson

## Abstract

The International Practical Temperature Scale of 1948 is a text revision of the International Temperature Scale of 1948, the numerical values of temperatures remaining the same. The adjective “Practical” was added to the name by the International Committee on Weights and Measures. The scale continues to be based upon six fixed and reproducible equilibrium temperatures to which values have been assigned, and upon the same interpolation formulas relating temperatures to the indications of specified measuring instruments. Some changes have been made in the text to make the scale more reproducible than its predecessor. The triple point of water, with the value 0.01 °C replaces the former ice point as a defining fixed point of the scale. It is also recommended that the zinc point, with the value 419.505 °C, be used instead of the sulfur point. The recommendations include new information that has become available since 1948.

An internationally accepted scale on which temperatures can be measured conveniently and accurately is necessary for science and industry. As early as 1911 the directors of the national laboratories of Germany, Great Britain, and the United States agreed to undertake the unification of the temperature scales in use in their respective countries. A practical scale, named the International Temperature Scale, was finally agreed upon, was recommended to the Seventh General Conference on Weights and Measures by its International Committee on Weights and Measures, and was adopted in 1927.^1^

The General Conference on Weights and Measures is the official international body now representing 36 nations that subscribe to the Treaty of the Meter. The General Conference normally meets every six years, and at those times may adopt recommendations submitted by the International Committee. The International Committee is the executive body elected by the General Conference. It consists of 18 scientists, only one from any one nation, and it normally meets every two years. The International Committee now has six advisory committees of specialists most of whom represent large national laboratories. The Advisory Committee on Thermometry was authorized in 1933 and first met in 1939.

In 1948 a revision of the International Temperature Scale was prepared by the Advisory Committee and proposed to the International Committee. The International Committee recommended this revision to the Ninth General Conference which adopted it.^2^ At this time the General Conference also adopted the designation of degree Celsius in place of degree Centigrade or Centesimal.^3^ The revised scale was designed to conform as nearly as practicable to the thermodynamic scale as then known, while incorporating certain refinements, based on experience, to make the scale more uniform and reproducible than its predecessor. In the revision there were only three changes which affected values of temperatures on the scale. One was to increase the value assigned to the silver point by 0.3 degree, merely to make the scale more uniform. Another was to specify Planck’s radiation formula instead of Wien’s formula so the scale would be consistent with the thermodynamic scale above the gold point. The third was to increase the value for the second radiation constant to bring it nearer to the value derived from atomic constants.

In 1954 the Advisory Committee proposed a resolution redefining the Kelvin thermodynamic scale by assigning a value to the triple point of water. This kind of definition was what Kelvin, in 1854, had said “must be adopted ultimately.” This resolution was recommended by the International Committee and adopted by the Tenth General Conference.^4^ As soon as this resolution had been adopted it was pointed out that it would be necessary to revise the introduction of the text of the International Temperature Scale of 1948 to conform with the action just taken.

In preparing a tentative proposal for a new text of the introduction it soon became evident that the other three parts of the text would also profit by a revision. For example, the triple point of water could now be made one of the defining fixed points of the scale and thus become the one defining fixed point common to both the international and the Kelvin scales. The Recommendations could include new information that had become available since 1948. At the higher temperatures some new determinations of differences between the international and thermodynamic scales could be included. The values reported for these differences, however, were still not certain enough to warrant a change of the scale itself. The new text, therefore, does not change the value of any temperature on the 1948 scale by as much as the experimental error of measurement.

In 1958 the tenative proposal was discussed in detail at sessions of the Advisory Committee in June, and many suggested changes were agreed upon. It was proposed to the International Committee in October. Minor corrections were made during the next two years, and in 1960 the International Committee gave the scale its new name. The International Committee recommended this text revision to the Eleventh General Conference and it was adopted in October 1960.

A translation of the official text 5 follows.

## 1. Introduction

The Kelvin thermodynamic scale, on which temperatures are designated as °K and denoted by the symbol *T*, is recognized as the fundamental scale to which all temperature measurements should ultimately be referable. The magnitude of the degree Kelvin is now defined by the decision (Tenth General Conference on Weights and Measures, 1954, Resolution 3) fixing the thermodynamic temperature of the triple point of water at exactly 273.16 °K.

The experimental difficulties inherent in the measurement of temperature on the thermodynamic scale led to the adoption in 1927, by the Seventh General Conference on Weights and Measures, of a practical scale called the “International Temperature Scale”. This scale was intended to be conveniently and accurately reproducible and to agree as closely as practicable with the thermodynamic scale.

The International Temperature Scale was revised in 1948 to make it conform with the state of the knowledge then available.

In May 1960 the International Committee on Weights and Measures approved the new name, “International Practical Temperature Scale of 1948”, for the scale presented in this document. Inasmuch as the numerical values of temperature on this scale are the same as in 1948, this scale is not a revision of the scale of 1948 but merely a revision of its text.

## 2. Definition of the International Practical Temperature Scale of 1948

Temperatures on the International Practical Temperature Scale of 1948 are expressed in degrees Celsius, designated by °C or °C (Int. 1948), and are denoted here by the symbol *t* or *t_int_*.

The International Practical Temperature Scale is based on six reproducible temperatures (defining fixed points), to which numerical values are assigned, and on formulas establishing the relation between temperature and the indications of instruments calibrated by means of values assigned to the six defining fixed points. These fixed points are defined by specified equilibrium states, each of which, except for the triple point of water, is under a pressure of 101 325 newtons/meter^2^ (1 standard atmosphere).

The fixed points of the scale and the exact numerical values assigned to them are given in table 1.

The procedures for interpolation lead to a division of the scale into four parts.

- From 0 °C to 630.5 °C (antimony point) the temperature *t* is defined by the formula
  $$R_{t}=R_{0}\left(1+At+Bt^{2}\right),$$
  where *R_t_* is the resistance at temperature *t* of the platinum wire resistor of a standard resistance thermometer, and *R*_0_ is the resistance at 0 °C. The constants *R*_0_,* A*, and *B* are to be determined from the values of *R_t_* at the triple point of water, at the steam point, and at the sulfur point (or the zinc point). The platinum wire of a standard resistance thermometer shall be annealed and its purity shall be such that *R*_100_*/R*_0_ is not less than 1.3920.
- From the oxygen point to 0 °C, the temperature *t* is defined by the formula
  $$R_{t}=R_{0}[1+At+Bt^{2}+C(t–t_{100})t^{3}],$$
  where *R*_0_,* A*, and *B* are determined in the same manner as in *a* above, the constant *C* is to be determined from the value of *R_t_* at the oxygen point, and *t*_100_ = 100 °C.
- From 630.5 °C to the gold point the temperature *t* is defined by the formula
  $$E=a+bt+ct^{2},$$
  where *E* is the electromotive force of a standard thermocouple of platinum and platinum-rhodium alloy, when one of the junctions is at 0 °C and the other at the temperature *t.* The constants *a*, *b*, and *c* are to be determined from the values of *E* at 630.5 °C, at the silver point, and at the gold point. The value of the electromotive force at 630.5 °C is to be determined by measuring this temperature with a standard resistance thermometer.
  The wires of the standard thermocouple shall be annealed and the purity of the platinum wire shall be such that the ratio *R*_100_/*R*_0_ is not less than 1.3920. The platinum-rhodium wire shall consist nominally of 90 percent platinum and 10 percent rhodium by weight. When one junction of the thermocouple is at 0 °C and the other is successively at 630.5 °C, the silver point, and the gold point, the completed thermocouple shall have electromotive forces such that
  $$\begin{array}{l} \;E_{\text{Au}}=10\;300\;\mu V\pm 50\;\mu V\\ \;E_{\text{Au}}-E_{\text{Ag}}=1183\;\mu V+0.158\;(E_{\text{Au}}-10\;300\;\mu V)\pm 4\;\mu V\\ E_{\text{Au}}-E_{630.5}=4766\;\mu V+0.631\;(E_{\text{Au}}-10\;300\;\mu V)\pm 8\;\mu V. \end{array}$$
- Above the gold point the temperature *t* is defined by the formula
  $$\frac{J_{t}}{J_{\text{Au}}}=\frac{\exp\left[\frac{C_{2}}{\lambda (t_{\text{Au}}+T_{0})}\right]-1}{\exp\left[\frac{C_{2}}{\lambda (t+T_{0})}\right]-1},$$
  where *J_t_* and *J*_Au_ are the radiant energies per unit wavelength interval at wavelength λ, emitted per unit time per unit solid angle per unit area of a black- body at the temperature *t* and the gold point respectively, *C*_2_ is the second radiation constant with the value *C*_2_=0.014 38 meter-degrees, λ is in meters, and *T*_0_=273.15 degrees.

## 3. Recommendations

The following recommendations are advisory rather than mandatory. The recommended apparatus, methods, and procedures represent good practice at the present time, but there is no intention of retarding the development and use of improvements and refinements. Experience has shown these recommendations to be in the interest of uniformity and reproducibility in the realization of the International Practical Temperature Scale defined in Section 2.

### 3.1. Standard Resistance Thermometer

A standard resistance thermometer should be so designed and constructed that the wire of the platinum resistor is as nearly strain free as practicable and will remain so during continued use. The platinum wire should be drawn from a fused ingot, not from forged sponge.

Standard resistance thermometers have been made of wire with diameters between 0.05 and 0.5 mm, at least a short portion of each lead adjacent to the resistor also being of platinum. The completed resistor of the thermometer should be annealed in air at a temperature higher than the highest temperature at which it is to be used, but in no case below 450 °C. There is reason to believe, furthermore, that better stability is obtained when the tube protecting the completed resistor is filled with gas containing some oxygen.

Useful criteria which serve as safeguards against inferior construction of the completed thermometer and against errors in the calibrations at the fixed points are that the value of the constant *B* is (−0.5857 ±0.0010) × 10^−6^/deg^2^ and that the value of the constant *C* is (−4.35±0.05)× 10^−12^/deg^4^. Another useful criterion of the adequacy of the annealing and of the reliability of the thermometer is the constancy of its resistance at some reference temperature. For example the resistance of a thermometer at the triple point of water should not change by as much as the equivalent of 0.001 deg when the thermometer is subjected to temperature cycles such as are necessary for its calibration.

### 3.2. Standard Thermocouple

Standard thermocouples have been made of wires having a diameter between 0.35 and 0.65 mm.

Before calibration the wires of the thermocouple should be carefully annealed to ensure the constancy of the electromotive forces during use. For this it is necessary to heat the platinum wire to a temperature of at least 1100 °C and the platinum-rhodium wire to 1450 °C. If the annealing is done before the wires are mounted in their insulators, the completed thermocouple should be heated again to a temperature of at least 1100 °C, until the electromotive force is stable and local inhomogeneities caused by strains have disappeared. When the thermocouple has been annealed sufficiently, its indications should not vary with a change in the temperature gradient along the wire; they should not vary, for example, with the depth of immersion in an enclosure at uniform temperature.

The electromotive force of the thermocouple at 630.5 °C should be determined from measurements at some uniform and constant temperature between 630.3 and 630.7 °C.

### 3.3. Pressure

In practice, pressures are determined with a mercury column. Pure mercury may be taken as having a mean density at 20 °C of 13 545.87 kg/m^3^ in a mercury column balancing one atmosphere. In the practical determination of the standard atmosphere the International Committee on Weights and Measures recommends that the value of local gravity be expressed in the Potsdam system until it sanctions the use of another system.

In the following sections on the oxygen point, the steam point, and the sulfur point the formulas for the equilibrium temperatures *t_p_* are given as polynomials in powers of (*p/p*_0_*−*1), where *p* is the equilibrium pressure and *p*_0_ is one standard atmosphere. The limits of accuracy are also given for stated ranges of pressure. In practice, the errors caused by using these formulas are less than the errors caused by the instability of systems open to the atmosphere. Greater stability and increased accuracy can be realized in closed systems held at a constant pressure which is maintained within a few parts in a thousand of one atmosphere. Only the first power terms of (*p*/*p*_0_−1) hi the polynomials are then needed.

### 3.4. Zero Point of the Scale and Triple Point of Water

#### a. Zero Point of the Scale

The zero point of the International Practical Temperature Scale is defined as being the temperature exactly 0.01 deg below the triple point of water. Calculations show that the temperature of the former “ice point”, defined as the temperature of equilibrium between ice and air-saturated water under a pressure of one standard atmosphere, is 0 °C within 0.0001 deg.

It is difficult, however, to obtain the ice point directly with an accuracy better than ±0.001 deg; but when this accuracy suffices, the temperature 0 °C may be be realized by the use of a mixture of finely divided ice and water saturated with air at 0 °C in a well insulated container such as a Dewar flask. The equilibrium temperature *t* corresponding to an ambient atmosphere pressure *p* and at a depth *h* below the surface of the water, may be calculated by the formula

$$t=0.01\;(1-p/p_{o})\;{}^{\circ}C-(0.7\times 10^{-6}\deg/\text{mm})h.$$

#### b. Triple Point of Water

The temperature of the triple point of water has been realized in sealed glass cells which contain only water of high purity; these cells have axial re-entrant wells for thermometers. In such cells the triple-point temperature is realized wherever ice is in equilibrium with a liquid-vapor surface. At a depth of *h* below the liquid-vapor surface the equilibrium temperature between ice and liquid water is given by the formula

$$t=0.01\;{}^{\circ}C-(0.7\times 10^{-6}\deg/\text{mm})h.$$

The recommended method of preparing a triple-point cell for use is first to freeze a thick mantle of ice around the well by cooling from within, and then to melt enough of this mantle, also from within, to produce a new water-ice interface close to the well. After these cells have been prepared for use, the temperatures measured in the wells have been found to rise by amounts ranging from 0.0001 to 0.0005 deg before becoming stable after from 1 to 3 days. This initial change can probably be explained either by the increase in the dimensions of the crystals of ice or by the slow release of strains in the crystals. A cell prepared in this manner and kept in an ice bath is capable of maintaining a temperature which is constant within about 0.0001 deg for several months. When cells from different sources have been compared under these conditions, no differences greater than 0.0002 deg have been reported.

Water from most natural sources (normal water) contains about 0.0148 mole percent deuterium, 0.20 mole percent O^18^, and 0.04 mole percent O^17^. Variations from this norm as large as 0.0015 mole percent have been found in the deuterium content of natural waters. An increase of 0.001 mole percent in the deuterium content of water corresponds to an increase of 0.000 04 deg in the triple-point temperature. Waters from rivers that rise to the leeward of mountain ranges or at the base of permanent glaciers may contain less than the normal amount of deuterium, whereas waters from the surface of large lakes may contain more than the normal amount.

The isotopic composition at the water-ice interfaces in the triple-point cells depends also on the natural differences in the proportions of the oxygen isotopes, on the process of distilling water, and on the procedure of freezing. The effects of these different isotopic compositions on the temperatures realized in triple-point cells are probably sufficiently small to be neglected.

### 3.5. Oxygen Point

The temperature of equilibrium between liquid oxygen and its vapor is usually realized by the static method. The platinum resistor of a standard thermometer and the liquid oxygen in its container are brought to the same temperature in a metal block placed in a suitable cryostat. The metal block is usually immersed in a well-stirred bath of liquid oxygen open to the atmosphere, but greater stability has been obtained by enclosing the metal block inside an evacuated envelope maintained at a uniform temperature near the oxygen point. The oxygen vapor pressure is transmitted through a tube leading out to a manometer. The entire length of this tube should be at temperatures above the saturation temperature of the oxygen.

Criteria that the equilibrium temperature has been realized are that the observed temperature, corrected to constant pressure at the free surface of the oxygen, is independent of: Small variations in the depth of immersion of the thermometer in the metal block, the ratio of the volume of the liquid oxygen to the volume of the vapor, and small variations in the temperature of the envelope.

The equilibrium temperature *t_p_* corresponding to a pressure *p* at the surface of the liquid oxygen may be found to an accuracy of a few thousandths of a degree over the range from *p*=660 mm to *p* = 860 mm of mercury by means of the formula

$$t_{p}=[-182.97+9.530(p/p_{o}-1)-3.72{\left(p/p_{o}-1\right)}^{2}+2.2{\left(p/p_{o}-1\right)}^{3}]{}^{\circ}C.$$

### 3.6. Steam Point

The temperature of equilibrium between liquid water and its vapor is usually realized by the dynamic method with the thermometer placed within the saturated vapor. Open systems were formerly used for the realization of the steam point but for precise calibration it is preferable to use a closed system in which a boiler and a manometer are connected to a manostat filled with air or, preferably, helium.

The boiler should be constructed so as to avoid all contamination of the vapor. The thermometer should be shielded against radiation from bodies that are at temperatures different from the saturation temperature.

The criteria that the equilibrium temperature has been realized are that the observed temperature, corrected to a constant pressure, is independent of: The water used, the elapsed time, the variations in the heat input to the liquid water, and the depth of immersion of the thermometer.

The equilibrium temperature *t_p_*, corresponding to a pressure *p*, may be found to within 0.001 deg over the range between *p*=660 mm and *p*=860 mm of mercury by means of the formula

$$t_{p}=[100+28.012(p/p_{o}-1)-11.64{\left(p/p_{o}-1\right)}^{2}+7.1{\left(p/p_{o}-1\right)}^{3}]{}^{\circ}C.$$

A change in the proportion of deuterium in water produces about one third as much change at the boiling-point temperature as that produced at the triple point, and in the same direction.

### 3.7. Sulfur Point

The temperature of equilibrium between liquid sulfur and its vapor is usually realized by the dynamic method in an aluminum boiler similar in shape to that used for the steam point except that extra shielding against radiation and larger spaces for free circulation of the vapor are needed.

The addition of 0.1 percent of arsenic and then of 0.1 percent of selenium to sulfur has been reported to raise the normal boiling point by 0.02 deg and then by 0.07 deg. These elements are common in sulfur from volcanic sources. Commercial sulfur contains organic impurities which slowly decompose and leave carbon when the sulfur is boiled. Carbon itself probably has no observable effect on the boiling point of sulfur, but it is preferable to remove the organic matter and carbon.

The criteria that the sulfur point has been realized are similar to those for the realization of the steam point, except that it may take many hours to reach a constant temperature.

The equilibrium temperature *t_p_*, corresponding to a pressure *p*, may be found to an accuracy of about 0.001 deg in the range between *p*= 660 mm and *p* = 800 mm of mercury by means of the formula

$$t_{p}=[444.6+69.010(p/p_{o}-1)-27.48{\left(p/p_{o}-1\right)}^{2}+19.14{\left(p/p_{o}-1\right)}^{3}]{}^{\circ}C.$$

### 3.8. Zinc Point

Highly reproducible temperatures, closely related to those which give the temperature of the liquidus curve of an alloy, have been realized as plateau temperatures on slow-rate freezing curves of high purity (99.999 weight percent) zinc.

The zinc is usually melted and frozen in high purity artificial graphite (99.999 weight percent) crucibles with axial thermometer wells, in simple block furnaces. The crucibles should be about 5 cm in diameter and deep enough to eliminate the effects of heat conduction along the thermometer leads.

When cooling has been started and solid has begun to form on the crucible wall, the thermometer should be removed, cooled to the room temperature, and then reinserted in its well to induce a thin mantle of solid zinc on the outside of the well. Another technique, which has been used, is to remove the thermometer when the temperature indicated by it is 0.01 deg below the freezing point, and to insert a silica rod for about 30 sec; the thermometer is then replaced in its well. The plateau temperature is that of equilibrium between the liquid zinc and the solid zinc of the mantle while freezing is progressing slowly inward from the outside of the crucible. The melts are best made in an inert atmosphere to inhibit oxidation of the graphite and zinc, yet there has been no evidence of the plateau temperatures being affected by zinc oxide in the melt even after prolonged heating in air. The plateau temperatures have been found to increase 0.0043 deg per atmosphere.

A criterion of adequate purity of a sample is that its melting range is not greater than about 0.001 deg. Samples of zinc of this high purity, originating in different countries, have given plateau temperatures which were practically identical (within 0.0002 deg). Samples with melting ranges of about 0.01 deg yielded plateau temperatures that were low by from 0.0004 deg to 0.0016 deg.

### 3.9. Silver and Gold Points

The temperature of equilibrium between solid and liquid silver or between solid and liquid gold has been realized in covered crucibles either of high purity artificial graphite, or ceramic, or vitreous silica. The dimensions of the crucibles should be such as to allow for the considerable expansion of these metals on melting and the crucibles should be deep enough to eliminate the effects of heat conduction along the thermocouple wires. Silver must be protected from oxygen while molten.

The crucible and its contents should be brought to a uniform temperature a few degrees above the melting point of the metal and then allowed to cool slowly. A thermocouple, mounted in a protecting tube of porcelain or other suitable material, with insulators separating the two wires, is immersed in the molten metal which is then allowed to freeze.

Criteria that the equilibrium temperature has been realized are: That the electromotive force of the thermocouple is independent of small changes in the depth of immersion during successive freezings, and that the electromotive force remains essentially constant for a period of at least 5 min during a single freezing.

For the range of the scale above 1063 °C, where the Planck radiation formula is used, the gold-point crucible should be modified to have a blackbody cavity at the temperature of the freezing gold.

## 4. Supplementary Information

### 4.1. Resistance—Temperature Formulas

The interpolation formula for the range 0 to 630.5 °C as given in the definition of the scale (sec. 2, *a*),

$$R_{t}=R_{0}(1+At+Bt^{2}),$$

may be written in the Callendar form

$$t=\frac{1}{\alpha }\left(\frac{R_{t}}{R_{0}}-1\right)+\delta \left(\frac{t}{t_{100}}-1\right)\frac{t}{t_{100}}$$

where

$$\alpha =\frac{1}{t_{100}}\left(\frac{R_{100}}{R_{0}}-1\right),\;\text{and}\;t_{100}=100{}^{\circ}C.$$

The relations between the coefficients are

$$\begin{array}{ll} A=\alpha \left(1+\frac{\delta }{t_{100}}\right), & \alpha =A+Bt_{100},\\ B=-\frac{\alpha \delta }{t_{100}^{2}}, & \delta =-\frac{Bt_{100}^{2}}{A+Bt_{100}}. \end{array}$$

The interpolation formula for the range 0°C to the oxygen point as given in the definition of the scale (sec. 2b),

$$R_{t}=R_{0}[1+At+Bt^{2}+C(t-t_{100})t^{3}],$$

may be written in the Callendar-Van Dusen form

$$t=\left[\frac{1}{\alpha }\left(\frac{R_{t}}{R_{0}}-1\right)+\delta \left(\frac{t}{t_{100}}-1\right)\frac{t}{t_{100}}+\beta \left(\frac{t}{t_{100}}-1\right){\left(\frac{t}{t_{100}}\right)}^{3}\right]{}^{\circ}C.$$

The relations connecting *A, B* and *α, δ* are the same as those given above and the other relations are

$$C=-\frac{\alpha \beta }{t_{100}^{4}},\;\beta =-\frac{Ct_{100}^{4}}{A+Bt_{100}}.$$

### 4.2. Secondary Reference Points

In addition to the defining fixed points of the scale, given in table 1, certain other points may be useful for reference purposes. Some of these and their corresponding reported temperatures on the International Practical Temperature Scale of 1948 are given in table 2. Except for the triple points, each temperature is for a system in equilibrium under a pressure of 1 standard atmosphere. The formulas for the variation of temperature with pressure are intended for use over the range of pressures from *p* = 680 mm to *p* =780 mm of mercury.

### 4.3. Relation Between the International Practical Temperature Scale and the Thermodynamic Scale

When the International Temperature Scale was adopted in 1927 it was in as close accord with the thermodynamic scale as was practicable with the knowledge then available. It was recognized, however, that further research would increase our knowledge of the actual differences between values of temperature on the two scales. When it is desired to know the value of a temperature on the thermodynamic scale, the usual procedure is to obtain the value on the International Practical Temperature Scale and then to convert it to the thermodynamic scale by adding the appropriate difference between the scales. These differences, however, have to be determined by experiment. They are difficult to determine accurately because they are small compared with their Kelvin temperature. Some of these differences obtained in various parts of the scale are given below in order to how the present state of our information about the agreement of the two scales.

On account of the uncertainties in these differences it seems preferable not to modify the values of temperature on the International Practical Temperature Scale now but to further improve our knowledge of the differences between the scales. When it is desired, it will be possible to improve the means of determining temperatures on the International Practical Temperature Scale without changing significantly the values of temperature. This procedure will avoid the confusion which would result from too frequent changes in values of temperature.

In the range from 0 °C to the sulfur point, intercomparisons of two nitrogen gas thermometers with standard resistance thermometers were reported in 1939 from The Massachusetts Institute of Technology. The differences found between the thermodynamic Celsius temperature *t_th_* (definition of 1954) and the temperature *t_int_* (1948 scale) have been formulated as follows:

$$t_{th}-t_{\mathrm{int}}=\frac{t}{t_{100}}\left[-0.0060+\left(\frac{t}{t_{100}}-1\right)(0.04106-7.363\times 10^{-5}\deg^{-1}t)\right]\deg.$$

This relation gives 99.994 °C (therm.) for the steam point and 444.70 °C (therm.) for the sulfur point. The results obtained with the two gas thermometers differed by 0.005 deg at the steam point and 0.05 deg at the sulfur point. In 1958 the PhysikalischTechnische Bundesanstalt reported the value 444.66 °C (therm.) for the sulfur point.

In the range from the oxygen point to 0 °C, investigations at the Physikalisch-Technische Reichsanstalt, reported in 1932, and at the University of Leiden, reported in 1935, give a set of values which indicate that the differences *t_th_*−*t_int_* have a maximum of about +0.04 deg at about −80 °C. Below −100 °C some of the reported differences have opposite signs. These differences are of the order of magnitude of the possible uncertainties in the gas thermometer measurements. For the oxygen point, the results published since 1927 by four laboratories have been recalculated on the basis of the value *T*_0_=273.15°K (adopted in 1954). These values are 90.191 °K from the Physikalisch-Technische Reichsanstalt (1932), 90.17 °K from the Tohoku University, Sendai, Japan (1935), 90.160 °K from the University of Leiden (1940), and 90.150 °K from the Pennsylvania State University (1953). The average of these four results is 90.168 °K or −182.982 °C (therm.).

The International Practical Temperature Scale is not defined below the oxygen point.

In the neighborhood of 1000 °C new determinations of the thermodynamic temperature of the silver point and gold point have been made in recent years in Germany and Japan. At the PhysikalischTechnische Bundesanstalt (1958) 962.16 °C (therm.) was obtained for the silver point and 1064.76 °C (therm.) for the gold point. At the Tokyo Institute of Technology (1958), the values 961.20 °C (therm.) and 1063.73 °C (therm.) were obtained for these points; these last two values being little different from those reported by the same laboratory in 1956, namely 961.28 °C (therm.) and 1063.69 °C (therm.)

In the range above the gold point the Planck radiation formula is used. The Planck formula is consistent with the thermodynamic scale and hence it would give true values of Kelvin temperature if the correct values were known for the Kelvin temperature of the gold point and for the second radiation constant *C*_2_.

An analysis of variance of data on atomic constants by The California Institute of Technology, published in 1955, gave the value 0.014 388 8 meter-degree for *C*_2_. A similar analysis from The Johns Hopkins University, published in 1957, gave the value 0.014 388 6 meter-degree for *C*_2_.

The international practical Kelvin temperatures are obtained by adding the value of *T*_0_ = 273.15 deg to the international practical Celsius temperatures, defined above. Values of the thermodynamic Celsius temperatures are obtained by subtracting *T*_0_ from the thermodynamic Kelvin temperatures. Table 3 gives the recommended designations; the arrows point from the defined temperatures to the temperatures derived by changing the origin.

## Figures and Tables

**Table 1:** Defining fixed points Exact values assigned. The pressure is 1 standard atmosphere, except for the triple point of water.

	*Temperature °C* (*Int. 1948*)
Temperature of equilibrium between liquid oxygen and its vapor (oxygen point)	−182.97
Temperature of equilibrium between ice, liquid water, and water vapor (triple point of water)	+ 0.01
Temperature of equilibrium between liquid water and its vapor (steam point)	100
Temperature of equilibrium between liquid sulfur and its vapor (sulfur point)	444.6*
Temperature of equilibrium between solid silver and liquid silver (silver point)	960.8
Temperature of equilibrium between solid gold and liquid gold (gold point)	1063

* In place of the sulfur point, it is recommended to use the temperature of equilibrium between solid zinc and liquid zinc (zinc point) with the value 419.505 °C (Int. 1948). The zinc point is more reproducible than the sulfur point and the value which is assigned to it has been so chosen that it use leads to the same values of temperature on the International Practical Temperature Scale as does the use of the sulfur point.

**Table 2:** Secondary reference points Under a pressure of 1 standard atmosphere, except for the triple points.

	*Temperature °C (Int. 1948)*
Temperature of equilibrium between solid carbon dioxide and its vapor	−78.5
*t_p_*=[−78.5 to +12.12(*p*/*p*_o_−1)−6.4(*p*/*p*_o_−1)^2^]°C	
Temperature of equilibrium between solid mercury and liquid mercury	−38.87
Temperature of equilibrium between ice and air-saturated water	0.000
Temperature of triple point of phenoxybenzene (diphenyl-ether)	26.88
Temperature of transition of sodium sulphate decahydrate	32.38
Temperature of triple point of benzoic acid	122.36
Temperature of equilibrium between solid indium and liquid indium	156.61
Temperature of equilibrium between liquid naphthalene and its vapor	218.0
*t_p_*[=218.0+44.4(*p*/*p*_o_−1) −19(*p*/*p*_o_−1)^2^]°C	
Temperature of equilibrium between solid tin and liquid tin	231.91
Temperature of equilibrium between liquid benzophenone and its vapor	305.9
*t_p_*+[305.9+48.8(*p*/*p*_o_−1) −21 (*p*/*p*_o_−1)^2^]°C	
Temperature of equilibrium between solid cadmium and liquid cadmium	321.03
Temperature of equilibrium between solid lead and liquid lead	327.3
Temperature of equilibrium between liquid mercury and its vapor	356.58
*t_p_* = [356.58+55.552(*p*/*p*_o_−1) −23.03(*p*/*p*_o_−1)^2^+14.0(*p*/*p*_o_−1)^3^]°C	
Temperature of equilibrium between solid aluminum and liquid aluminum	660.1
Temperature of equilibrium between solid copper and liquid copper (in a reducing atmosphere)	1083
Temperature of equilibrium between solid nickel and liquid nickel	1453
Temperature of equilibrium between solid cobalt and liquid cobalt	1492
Temperature of equilibrium between solid palladium and liquid palladium	1552
Temperature of equilibrium between solid platinum and liquid platinum	1769
Temperature of equilibrium between solid rhodium and liquid rhodium	1960
Temperature of equilibrium between solid iridium and liquid iridium	2443
Temperature of melting tungsten	3380

**Table 3:** 

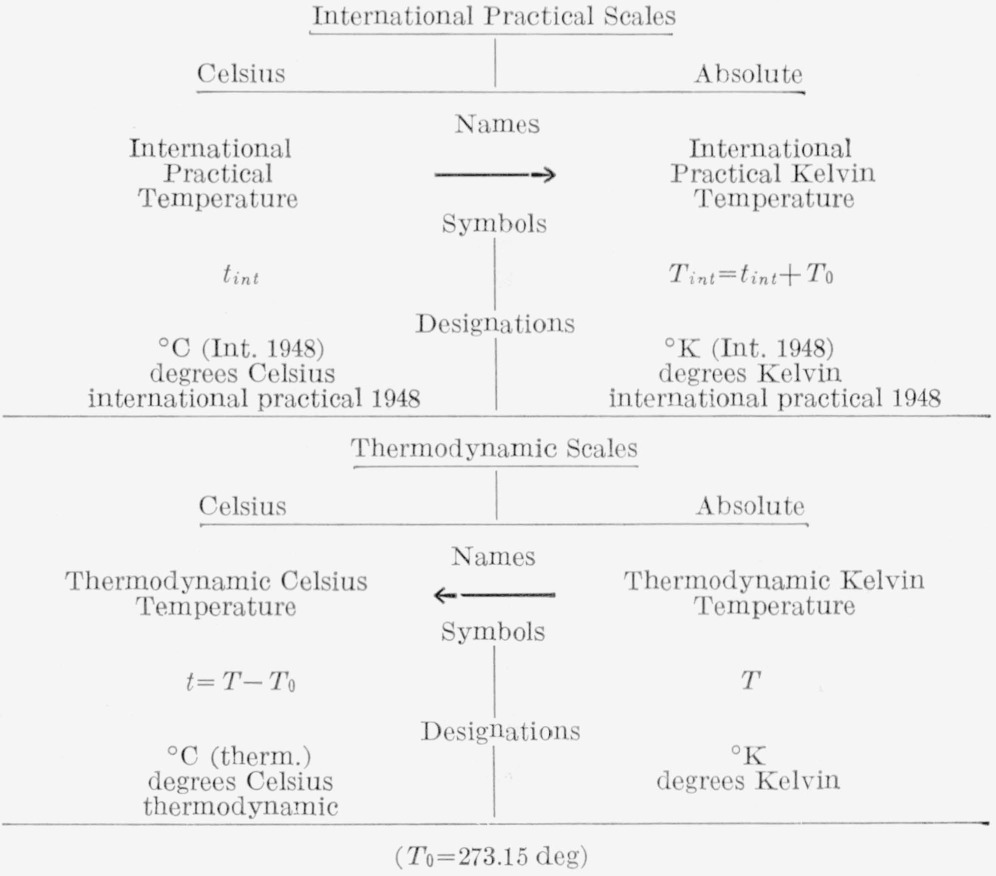

Note. For the international practical temperature, the subscript “int” after *t* may be omitted if there is no possibility of confusion.

## Footnotes

1 Comptes Rendus de la Septième Conférence Générale des Poids et Mesures, p. 94 (1927).

2 Comptes Rendus de la Neuvième Conférence Générale des Poids et Mesures, p. 89 (1948).

3 Comptes Rendus de la Neuvième Conférence Générale des Poids et Mesures, p. 64 (1948).

4 Comptes Rendus de la Dixième Conférence Générale des Poids et Measures, p. 79 (1954).

5 Comptes Rendus de la Onzième Conférence Générale des Poids et Mesures (1960).

